# Spectrum of Disorders of Sex Development: A Single-Center Experience in the Southern Area of Iraq

**DOI:** 10.7759/cureus.67571

**Published:** 2024-08-23

**Authors:** Nazar F Al-Sahar, Ahmad J Al-Ali, Abbas A Mansour

**Affiliations:** 1 Endocrinology/Diabetes/Metabolism, Faiha Specialized Diabetes, Endocrine and Metabolism Center (FDEMC) University of Basrah, Basrah, IRQ; 2 Pediatric Endocrinology, Faiha Specialized Diabetes, Endocrine and Metabolism Center (FDEMC) University of Basrah, Basrah, IRQ; 3 Diabetes and Endocrinology, Faiha Specialized Diabetes, Endocrine and Metabolism Center (FDEMC) University of Basrah, Basrah, IRQ

**Keywords:** disorders of sex development, atypical genitalia, 46xy dsd, 46xx dsd, turner, karyotype

## Abstract

Background: The most common presentation of disorders of sex development (DSD) is in the neonatal period when a baby is born with atypical ("ambiguous") genitalia, making it unclear whether the child is a boy or a girl. This study aims to provide an overview of the DSD spectrum, seen in Faiha Specialized Diabetes, Endocrine and Metabolism Center (FDEMC), Basrah, southern area of Iraq.

Methods: A retrospective study on patients with DSD was referred to FDEMC, a tertiary center in Basrah, between January 2009 and December 2023.

Results: Out of the total 150 studied patients, individuals above 15 years old comprised the majority. Sex chromosomal DSD made up 37.3% of the cases, while 46, XY DSD comprised 34.7%, and 46, XX DSD accounted for 28% of the total.

Conclusion: Many patients with DSD in Basrah were diagnosed late, beyond infancy. Increasing awareness among healthcare providers and families is essential for early diagnosis during infancy.

## Introduction

The interest in disorders of sex development (DSD) began to grow following the release of the Chicago consensus statement in 2006 [1]. After nine years, the Society for Endocrinology UK released the revised guidance in 2015 on the initial evaluation of infants or adolescents suspected to have a DSD, offering new insights [2], which was updated in 2021 [3]. These consensus statements make the use of intersex obsolete and open the era to new terminology, which is called DSD, i.e., differences of sex development, and disorders of sexual differentiation.

The potential advantages of this terminology lie in its medical precision and its ability to eliminate ambiguity, particularly by avoiding confusion with terms, such as gender dysphoria, transgenderism, and homosexuality. However, patients may perceive social stigma associated with DSD terminologies due to the connotations of "disorder" and the assumption that "sex" solely pertains to sexual activity [4].

DSD is a medical condition that encompasses a diverse group of congenital conditions characterized by the atypical development of chromosomal, gonadal, or anatomical sex. These conditions challenge conventional binary concepts of sex and gender by presenting a spectrum of variations that may not neatly align with typical male or female categorizations. DSD can manifest in various forms, including differences in genitalia, gonadal development, hormone levels, and chromosomal patterns [1].

The comprehension and treatment of DSD have undergone substantial evolution in recent years. There has been a transition from a primarily binary and pathologizing approach to one emphasizing individualized care, informed decision-making, and support for both affected individuals and their families. Advances in medical genetics, imaging methods, and psychosocial assistance have contributed to a more comprehensive understanding and holistic management of DSD [1].

Despite these advancements, navigating the complex medical, ethical, and social dimensions of DSD still presents challenges. Issues such as informed consent, appropriate medical interventions, psychosocial well-being, and advocacy for rights persist as key considerations shaping discussions and approaches to care for individuals affected by DSD [5].

When the gonad undergoes specialization due to the influence of sex-determining genes, the resulting phenotypic sex emerges from the interplay of distinct hormone actions on the differentiation of inner ducts and outer genitalia [6].

The most common presentation of DSD is in the neonatal period when a baby is born with atypical ("ambiguous") genitalia, making it unclear whether the child is a boy or a girl. In other situations, DSD can be diagnosed: following a mismatch between prenatal karyotype and phenotype; due to associated features in childhood (e.g., renal abnormalities and inguinal hernias); during adolescence with absent puberty, unexpected virilization, estrogenization, or primary amenorrhea; and sometimes in adulthood with infertility or as an incidental finding. These diverse presentations underscore the complexity of DSD and the need for careful evaluation and support for affected individuals and their families [7].

According to the Chicago consensus, DSD can be classified into three groups depending on karyotype and pathogenesis: sex chromosome DSD, XY DSD, and XX DSD [1].

This study aims to provide an overview of the DSD spectrum, seen in Faiha Specialized Diabetes, Endocrine and Metabolism Center (FDEMC), Basrah, Southern Iraq.

## Materials and methods

This is a retrospective study on patients with DSD referred to FDEMC, a tertiary center in Basrah, a southern area of Iraq, between January 2009 and December 2023.

FDEMC received patients with DSD from Basrah and other governorates from Iraq. In our country, the pediatric age group is defined as 14 years and below, with patients in this group being treated by pediatric endocrinologists. Patients older than 14 years are managed by adult endocrinologists.

**Figure 1:**
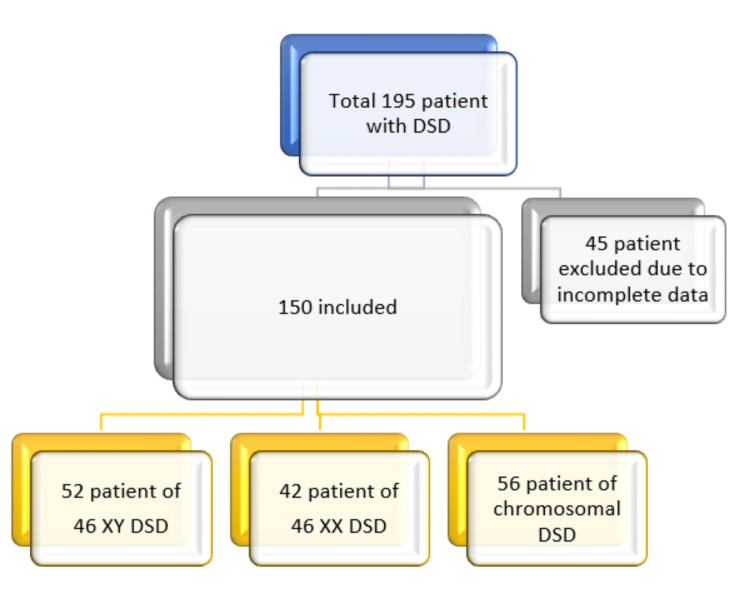
Database of the patients enrolled in the study.

Individual records of 195 patients who attended FDEMC were reviewed. Forty-five were excluded due to incomplete data (missed karyotype, hormonal, or other laboratory test or no obvious /clear final diagnosis), and only 150 case records were included in our study (Figure 1).

Inclusion criteria

The study included all patients with DSD who had complete medical records. The work-up was applied to cases with genital ambiguity, apparent male genitalia with non-palpable testes, severe hypospadias and/or bifid scrotum, hypospadias with undescended testes, apparent female genitalia with clitoral hypertrophy, posterior labial fusion, and inguinal/labial mass.

Exclusion criteria

Patients with incomplete data and those with atypical genitalia due to hypopituitarism, meatal stenosis, isolated sub-coronal hypospadias, isolated unilateral cryptorchidism with normal testicular function, and vesical exstrophy were excluded. In addition, patients with delayed puberty (absence of secondary sexual maturation by the age of 13 years in girls and 14 years in boys) due to systemic diseases (e.g., thalassemia and haemochromatosis) were also excluded from the study.

Participants

Key data obtained from the records included main complaints, demographic information (age, sex, and address), clinical presentation, age and mode of first presentation, sex of rearing, and diagnostic workup, including hormonal profiles, imaging studies, karyotype, and histopathological findings if available. The study utilized a multidisciplinary evaluation approach involving pediatric endocrinologists, geneticists, adult endocrinologists, urologists, and psychiatrists.

The patients were divided into three categories, namely, 46, XY DSD, 46, XX DSD, and sex chromosomal DSD, based on the available data and according to the Chicago Consensus in 2006 [1].

A thorough male genital examination was conducted for apparently male patients, assessing testicular volume (by Prader orchidometer), number, and location, along with scrotal examination and measurements of the penile length and the site of the metal orifice (opening). For apparently female patients, the external genitalia were examined for clitoral size, labial configuration, urethral orifice, and vaginal opening. In addition, secondary sexual characteristics were evaluated in both male and female patients.

The laboratory evaluation included hormonal assays (17 OH progesterone, testosterone, estradiol, follicle-stimulating hormone (FSH), luteinizing hormone (LH), cortisol, dehydroepiandrosterone-sulfate (DHEA-S), prolactin, thyroid-stimulating hormone (TSH), thyroxine (T4), and androstenediones) and biochemical assays. Chromosomal analysis was performed using standard methods. Ultrasound and magnetic resonance imaging (MRI) were used to evaluate internal genital structures, including the gonads. In addition, diagnostic laparoscopy was performed in some cases.

Definitions

Turner syndrome is defined by the presence of characteristic physical features in phenotypic females, typically including short stature or pubertal delay. It is often accompanied by markedly elevated levels of FSH and may involve complete or partial absence of secondary sexual characteristics. Diagnosis is confirmed through karyotyping, which typically reveals abnormalities such as monosomy X (45,X) or other chromosomal variations [8].

Klinefelter syndrome was diagnosed in males with tall stature, gynecomastia, eunuchoidism, small firm testis, and azoospermia with typical karyotype [9]. 46, XX DSD are subdivided into disorders of gonadal (ovarian) development and androgen excess. Congenital adrenal hyperplasia (CAH) diagnosis was made according to the guidelines [10]. Ovotesticular DSD, gonadal dysgenesis, androgen insensitivity syndrome, and 5 alpha-reductase deficiency diagnoses were made according to a previous report [11].

Statistical analysis

The patients’ data were entered into an Excel sheet (Microsoft Corporation, USA) and transformed into IBM SPSS Statistics for Windows, Version 23.0 (released 2014, IBM Corp., Armonk, NY). The continuous variable was expressed as mean ± SD, and categorical values were expressed as numbers and percentages. 

## Results

In Table 1, out of the total 150 studied patients, Individuals above 15 years old comprised the largest group, constituting 36.1% of the total sample; patients under one year old constituted 10.6%; patients aged one to five years represented 22%; and patients aged six to 14 years accounted for 31.4% of the total studied patients. In terms of gender, the majority of the patients identified as females, comprising 68% of the total patients, while males constitute 32%. Regarding geographical distribution, the highest proportion of patients reside in Basrah, constituting 82.7% of the total population. Other patients were from Nasiriyah (5.3%), Amarah (4%), Samawah (3.3%), Najaf (1.3%), Kirkuk (1.3%), Karbala (0.7%), and Babil (0.7%).

**Table 1:** Demographic characteristics among 150 patients with disorders of sex development.

Variables		No. (%)
Age at presentation	<1 year	16 (10.6)
	1-5 years	33 (22)
	6-14 years	47 (31.3)
	15 years or above	54(36.1)
Gender	Female	102 (68)
	Male	48 (32)
Address	Basrah	124 (82.7)
	Nasiriyah	8 (5.3)
	Amarah	6 (4)
	Samawah	5 (3.3)
	Najaf	2 (1.3)
	Karbala	1 (0.7)
	Babil	1 (0.7)
	Kirkuk	2 (1.3)
	Diwaniyah	1 (0.7)

In Table 2, the DSD classification is as follows: sex chromosomal DSD accounted for 56 patients, which is 37.3% of the total; 46, XY DSD was found in 52 patients, representing 34.7%; and 46, XX DSD was observed in 42 patients, comprising 28% of the total.

**Table 2:** Disorders of sex development (DSD) classification.

Class	Number (%)
46, XY DSD	52 (34.7%)
46, XX DSD	42 (28%)
Sex chromosomal DSD	56 (37.3%)
Total	150 (100%)

In Table 3, the distribution of DSD conditions among 46, XY individuals is as follows: androgen insensitivity syndrome (AIS) represented the most common diagnosis, with 31/52 patients (59.6%); 5-alpha reductase deficiency was observed in 7/52 patients (13.5%); CAH accounted for 7/52 patients (13.5%); and 46, XY gonadal dysgenesis was diagnosed in another 7/52 patients (13.5%).

**Table 3:** Final diagnosis and frequency of 150 patients with DSD. CAH: congenital adrenal hyperplasia, DSD: disorders of sex development *Of the total from each row. **3-beta hydroxysteroid dehydrogenase deficiency (three patients), 17-alpha hydroxylase deficiency (four patients). ***29 out of 38 patients (76.3 %) have karyotype (45 X0), and nine (23.6%) have (45X,46XX) mosaic karyotype.

Class	Diagnosis	Number (%*)
46, XY DSD	Androgen insensitivity syndrome	31 (59.6)
	5-Alpha reductase deficiency	7 (13.5)
	CAH (rare)**	7 (13.5)
	Gonadal dysgenesis	7 (13.5)
46, XX DSD	Classic CAH	33 (78.6)
	Gonadal dysgenesis	6 (14.3)
	Ovotesticular DSD	3 (7.1)
Sex chromosomal DSD	Turner*** syndrome	38 (67.9)
	Klinefelter syndrome	15 (26.8)
	Mixed gonadal dysgenesis	3 (5.4)
Total		150 (100)

Among the 46, XX DSD patients, classic CAH was seen in 33/42 patients, accounting for 78.6% of the total; 46, XX gonadal dysgenesis was observed in 6/42 patients, representing 14.3%; and ovotesticular DSD was diagnosed in 3/42 patients, comprising 7.1%.

Among the patients with sex chromosomal DSD, Turner syndrome comprised the majority of patients, totaling 38/56 (67.9%); among the Turner syndrome patients, 29 (76.3%) had the classic karyotype (45, X0) and nine patients (23.6%) had the mosaic karyotype (45, X/46, XX); Klinefelter syndrome was diagnosed in 15/56 patients, accounting for 26.8%; mixed gonadal dysgenesis was identified in 3/56 patients, comprising 5.4% of the sex chromosomal DSD cases.

In Table 4, the clinical features and management of patients with DSD are detailed, including genitalia appearance (description of the external genitalia phenotype), addressing any ambiguity or atypical features; surgical interventions (details of surgical procedures performed, such as genital reconstructive surgery or gonadectomy); documentation of additional health conditions observed in patients with DSD, such as infertility or stature abnormalities; and gender assignment variability (information on the process and decisions regarding gender assignment and rearing, reflecting the diverse approaches taken based on individual patient needs and preferences). This table provides a comprehensive overview of the clinical complexities and management strategies involved in caring for patients with DSD.

**Table 4:** Presentation of patients with DSD. DSD: disorders of sex development, NA: not applicable

		46, XY DSD	46, XX DSD	Turner syndrome	Klinefelter syndrome	
						Atypical genitalia	Yes	31 (59.6)	32 (76.2)	NA	NA	
No	21 (40.4)	10 (23.8)				
Virilization	Yes	2 (3.8)	19 (45.2)	NA	NA	
	No	50 (96.2)	23 (54.8)			
Undescended testes	Yes	26 (50.0)	1 (2.4)	NA	NA	
	No	26 (50.0)	41 (97.6)			
Gonadectomy	Yes	4 (7.7)	5 (11.9)	NA	NA	
	No	48 (92.3)	37 (88.1)			
Genitoplasty	Yes	5 (9.6)	9 (21.4)	NA	NA	
	No	47 (90.4)	33 (78.6)			
Gender reassignment	Yes	1 (1.9)	2 (4.8)	NA	NA	
	No	51 (98.1)	40 (95.2)			
Primary amenorrhea	Yes	12 (23.1)	4 (9.5)	15 (39.4)	NA	
	No	40 (76.9)	38 (90.5)	23 (60.6)		
Short stature	Yes	No data available	No data	35 (92.1)	NA	
	No			3 (7.9)		
Tall stature	Yes	No data available	No data	NA	15 (100.0)	
	No					
Gynecomastia	Yes	No data available	No data	NA	9 (60.0)	
	No				6 (40.0)	
Primary infertility	Yes	No data available	No data	No data	12 (80.0)	
	No				3 (20.0)	
Gender of rearing	Male	23 (44.2)	10 (23.8)			
	Female	29 (55.8)	32(76.2)			

For the patients with XY DSD, the clinical presentation and management details are summarized as follows: 31 patients (59.6%) presented with atypical genitalia; two patients (3.8%) presented with virilization; 26 patients (50%) presented with undescended testes; 12 patients (23.1%) presented with primary amenorrhea; four patients (7.7%) underwent gonadectomy; five patients (9.6%) underwent genitoplasty; and gender reassignment was performed in one patient (1.9%). These details highlight the varied clinical manifestations and management approaches involved in treating patients with XY DSD, addressing both physical and gender-related aspects as appropriate for each individual case.

Patients with 46, XX DSD presented with the following clinical features and management details: 32 patients (76.2%) presented with atypical genitalia; 19 patients (45.2%) presented with virilization; one patient (2.4%) had undescended testis; four patients (9.5%) presented with primary amenorrhea; gonadectomy was performed in five patients (11.9%); genitoplasty was performed in nine patients (21.4%); and gender reassignment was performed in two patients (4.2%).

In terms of gender assignment, 29 patients (55.8%) with 46, XY DSD were raised as girls, and 10 patients (23.8%) with 46, XX DSD were raised as boys.

All patients with Klinefelter syndrome presented with tall stature; of them, 12 (80%) had primary infertility and nine (60%) had gynecomastia. Regarding Turner syndrome, the majority of them presented with short stature (35, 92.1%), and 15 (39.4%) of them presented with primary amenorrhea.

## Discussion

This is the second study of its type in Iraq. DSD is a neglected topic in developing countries for a lot of factors like cultural, religious, and social and lack of experience.

The age of presentation of DSD depends upon the severity of the condition, family orientation, and awareness of the physician or nurse. In the current study, about one-third of patients presented in the adult age group (15 years and above), and a minority presented with an age less than one year. In the Duhok study done in the north of Iraq, which was a prospective and karyotyping-based classification, laparoscopic-based internal organ diagnosis and an abdominal ultrasound to diagnose DSD in the target population included 40 patients less than six years old over five years duration and the majority of patients presented at age three to five, which is preschool age [12].

In a retrospective study done in Cairo, which included 908 patients with DSD over a period of 43 years (1966-2009), the most common ages of presentation were adolescence and adulthood comparable to our results [13].

In a recently published study conducted in Turkey, between the years 2002 and 2018, which included 289 patients, the median age of the patients was two years [14].

In a UK study, which is a single-center retrospective, observational study of 607 children and young people referred to a DSD multidisciplinary team over 25 years (1995-2019), more than three-quarters of the patients were presented as neonates [7].

In a study done in Karachi, Pakistan, involving 48 patients with DSD between 2012 and 2014, the median age was 19 ± 8 [15]. It appears that there is a delay in the diagnosis of DSD in the current study, similar to findings observed in studies conducted in Cairo and Karachi [13,15]. This delay may reflect challenges in the early recognition and appropriate diagnostic evaluation of DSD, which are critical for timely management and support for affected individuals. Identifying and addressing these delays is crucial for improving outcomes and quality of life for patients with DSD.

Females are the most common presenting sex in more than two-thirds of patients with DSD involved in this study. In this study, the predominance of females as the presenting sex is similar to findings in the Duhok study, where more than half of the patients were female, and in the Karachi study, where less than two-thirds were female [12,15]. However, this differs from findings in the Cairo and UK studies, where the male gender was predominant in each study [7,13]. These variations highlight the geographical and population-specific differences in the distribution of sex presentation among patients with DSD in addition to differences in referral patterns and management practices.

In some regions or healthcare systems, there may be a higher threshold or differing criteria for referring cases of penoscrotal hypospadias or other forms of DSD to specialist services. This can result in a higher proportion of such cases being managed initially by plastic surgeons or general urologists rather than being referred to dedicated DSD centers. The availability of specialists trained in managing DSD, such as pediatric endocrinologists, urologists with expertise in DSD, and geneticists, can influence referral patterns. Regions with more specialized services for DSD may see a higher proportion of cases referred early to these specialists, influencing the demographic makeup of patients studied in research.

Our data show that sex chromosomal DSD was the most common DSD in the studied patients; it comprises about more than one-third. On the other hand, the Dohuk study, Turkish study, and the UK study all showed that sex chromosomal DSD was the least common [7,12,14]. This variability can be attributed to their study not encompassing classic Turner syndrome, as many patients with classic Turner syndrome were not referred through the DSD multidisciplinary team pathway, similar to findings in the UK study [7]. Alternatively, differences in referral pathways could also contribute to these variations. By contrast, both the Cairo and Karachi studies reported that sex chromosomal DSD was the most common, but in fewer than half of the patients [13,15].

In our study, 46, XY DSD were observed in one-third of the cases. Comparatively, in the Duhok study, half of the patients had 46, XY DSD [12], while in the Cairo study, it was less than one-third [13]. The Turkish study predominantly had 46, XY DSD, accounting for about half of the cases, whereas in the Karachi study, it was less than one quarter [14,15]. In the UK study, more than half of the cases were 46, XY DSD [7].

In our study, less than one-third of the cases involved were 46, XX DSD, whereas in the Duhok study, it was less than half [12]. The Karachi study reported about one-quarter, the UK study more than one-quarter, the Turkish study less than a quarter, and the Cairo study about one-third [7,13-15].

By contrast, 46, XY DSD was more common than 46, XX DSD in our study, aligning with findings from the Duhok, Turkish, and UK studies [7,12,14].

Among the patients with 46, XY DSD, androgen insensitivity syndrome (AIS) was the most frequent clinical diagnosis, observed in more than half of the cases. By contrast, 5-alpha reductase deficiency, congenital adrenal hyperplasia (CAH), and 46, XY gonadal dysgenesis were less common, consistent with findings from other studies such as the Duhok study, where AIS accounted for more than one-third, and the Turkish study, where it comprised about less than one-third of cases [12,14].

In patients with 46, XX DSD, classic CAH was the predominant diagnosis, accounting for more than two-thirds of cases. This pattern mirrors findings from the Duhok study [12], Turkish study [14], UK study [7], and Karachi study [15]. Gonadal dysgenesis and ovotesticular DSD were less frequently observed.

Turner syndrome was the predominant diagnosis among all the patients studied, comprising approximately one-quarter of the total cohort and about half of the cases of sex chromosomal DSD. This finding aligns with the Cairo study, where Turner syndrome was also the primary diagnosis, identified in more than a quarter of all patients studied [13]. In the Turkey study, Turner syndrome accounted for one-fifth of all patients studied and more than three-quarters of sex chromosomal DSD cases [14]. By contrast, Turner syndrome was less prevalent in the Dohuk, UK, and Karachi studies, possibly due to variations in referral practices or smaller sample sizes in those studies [7,12,15].

In our study, atypical genitalia were present in more than three-quarters of patients with 46, XX DSD, compared to less than two-thirds in 46, XY DSD, which represents more than one-third of the total cohort. Atypical genitalia were a predominant presentation in the Dohuk study, observed in more than two-thirds of cases [12].

In the Turkey study, more than one-quarter of patients with 46, XX DSD and less than one-fifth of those with 46, XY DSD presented with atypical genitalia [14]. In the UK study, less than two-thirds of all patients had atypical genitalia as a major presentation [7]. By contrast, the Cairo study reported that only one-tenth of patients presented with atypical genitalia [13]. However, in the current study and in each of the Dohuk study [12], Turkey study [14], Karachi study [15], and UK study [7], atypical genitalia were the primary presentation, complaint, or reason for referral.

Undescended testes were observed in half of the patients with 46, XY DSD and in only one patient with 46, XX DSD. In the Dohuk study, approximately one-quarter of the patients had undescended testes [12]. In the Turkey study, less than one-fifth of 46, XY DSD cases presented with undescended testes [14]. By contrast, in the UK study, undescended testes were a minority presentation, and this was similarly observed in the Karachi study [7,15].

Gender reassignment was infrequent in our cohort due to religious and social considerations [16,17].

Regarding the gender of rearing of our studied patients with 46, XY DSD, more than half were reared as female. However, in the Al-Jurayyan study conducted in Saudi Arabia, which was a retrospective study at a pediatric endocrine clinic from 1989 to 2008, only a minority of 46, XY DSD cases were assigned as female [18]. This variability can be attributed to differences in sample size and the presentation of our patients, many of whom presented with severe undervirilization due to defects in either androgen action or production.

In another study conducted in Egypt, 40 patients with 46, XY DSD were referred to the clinical genetics department at the National Research Center during the period 2006-2009. This study, the first of its kind in Egypt to investigate gender outcomes in patients with 46, XY DSD, revealed that more than three-quarters of the patients were assigned as female [19].

While in 46, XX DSD patients of our study, less than one-quarter were reared as male, an Indian retrospective study from a tertiary care hospital in North India, which included 194 DSD patients between 1995 and 2014, reported that about one-fourth of patients with CAH were reared as male [20].

In the Al-Jurayyan study conducted in Saudi Arabia, less than half of the 46, XX DSD patients were assigned as male [18]. This variability can be attributed to differences in sample size and the degree of virilization at presentation.

Primary amenorrhea was the primary presentation in less than a quarter of patients with 46, XY DSD, in less than half of patients with Turner syndrome, and in a minority of patients with 46, XX DSD.

In a Turkey study, a minority of 46, XY DSD and one-fifth of 46, XX DSD presented with primary amenorrhea [14].

In both the Cairo and Karachi studies, primary amenorrhea was observed in more than a quarter of female sex-assigned patients [13,15]. However, neither the Dohuk nor the UK study reported primary amenorrhea: the Dohuk study possibly due to the young age of the sample and the UK study possibly due to early diagnosis of DSD [7,12].

Limitations

The major limitations of this study were the retrospective design, lack of history of consanguinity, the small number of patients, and lack of a genetic study.

## Conclusions

Sex chromosomal DSD was the most prevalent DSD in this study, followed by XY DSD and XX DSD. However, many patients were diagnosed late, beyond infancy. There is a critical need to raise awareness about this condition among healthcare providers and families. Raising awareness involves addressing several key issues, including implementing educational programs and training medical staff on DSD as part of a multidisciplinary team framework. In addition, it is vital to enhance diagnostic infrastructure, promote early referral for suspected cases, and strengthen support systems for affected individuals and their families. Awareness campaigns targeted at families are also essential in this effort. It is also likely that DSD cases are underreported in Iraq.
